# A New MIMU/GNSS Ultra-Tightly Coupled Integration Architecture for Mitigating Abrupt Changes of Frequency Tracking Errors

**DOI:** 10.3390/mi11121117

**Published:** 2020-12-16

**Authors:** Shiming Liu, Sihai Li, Qiangwen Fu, Yuanbo Tao, Feng Wu

**Affiliations:** 1 School of Automation, Northwestern Polytechnical University, Xi’an 710072, China; lisihai@nwpu.edu.cn (S.L.); fuqiangwen@nwpu.edu.cn (Q.F.); 2 China Ordnance Industry Navigation and Control Technology Research Institute, Beijing 100089, China; taoyuanbo1990@mail.nwpu.edu.cn; 3 Shanghai Aerospace Control Technology Institute, Shanghai 201109, China; wufeng_nwpu@163.com

**Keywords:** global navigation satellite system (GNSS), ultra-tightly coupled integration, large frequency tracking errors, adaptive Kalman filter

## Abstract

We present a new ultra-tightly coupled (UTC) integration architecture of a micro-electromechanical inertial measurement unit (MIMU) and global navigation satellite system (GNSS) to reduce the performance degradation caused by abrupt changes of frequency tracking errors. A large frequency error will lead to a decrease in the carrier-to-noise ratio (*C*/*N*_0_) estimate and an increase in the code discriminator estimation error. The disruptive effects of frequency errors on the estimation of *C*/*N*_0_ and on the code discriminator are quantitatively evaluated via theoretical analyses and Monte Carlo simulations. The new MIMU/GNSS UTC architecture introduces a large frequency error detector and a refined frequency processor based on a retuned frequency in each tracking channel. In addition, an adaptive channel prefilter with multiple fading factors is introduced as an alternate to the conventional prefilter. Numerical simulations based on a highly dynamic trajectory are used to assess performance. The simulation results show that when there is an abrupt step change in the frequency tracking error, the new UTC architecture can effectively suppress the divergence of navigation solutions and the loss of tracking lock, and can significantly reduce the deviation of the *C*/*N*_0_ estimation.

## 1. Introduction

An inertial navigation system (INS) can be combined with multiple navigation technologies to form an integrated navigation system. Due to complementary error characteristics, INS is most commonly integrated with the global navigation satellite system (GNSS) [1]. INS/GNSS ultra-tightly coupled (UTC) integration navigation is an optimal INS/GNSS integrated navigation architecture [2]. The deep fusion between the INS and the receiver tracking loops greatly improves the highly dynamic adaptability and anti-interference ability. With the advantages of cost and size, the micro-electromechanical inertial measurement unit (MIMU) has been widely used in UTC navigation systems [3,4,5,6].

In the MIMU/GNSS UTC integration system, vector tracking loops (VTLs) are exploited to track the pseudocode phase and the carrier Doppler shift [7]. Under normal conditions, the tracking errors of VTLs are nominal. However, in some cases, especially in the highly dynamic context of maneuvering vehicles, the frequency tracking may exhibit large step deviations, leading to increased navigation errors and even loss of tracking lock. In the context of MIMU/GNSS UTC integration, there are two main reasons for the large jumps in frequency tracking errors.

One of the triggers is the crystal oscillator of the receiver. “Micro-jump” events, which refer to step changes in the local oscillation frequency, are well-known defects of crystal oscillators [8,9]. A “micro-jump” event is disruptive to the frequency tracking loops, which causes the local replica carrier frequency to deviate from its nominal value and results in a large jump in the frequency tracking error. In some extreme cases, a micro jump of as little as 20 parts per billion (ppb) can be sufficient to cause a loss of tracking [9].

The acceleration sensitivity or g-sensitivity of crystal oscillators will also induce a large frequency offset error [10,11]. A “2 g-tipover” test is a basic way to assess the g-sensitivity of crystal oscillators. The acceleration sensitivity can be compensated using IMU measurements to reduce the negative impacts on the GNSS receiver [12]. This method has better compensation effects in pedestrian and vehicle users, but its applicability in highly dynamic environments needs to be further evaluated.

MIMU is another component that induces large changes in frequency tracking errors. A low-cost MIMU is more prone to failure in highly dynamic environments. Some failures can make MIMU measurements unavailable. However, under multiple failure conditions, the MIMU is still accessible, but the measurement errors will exhibit step changes [13]. After integration operations, large specific force measurement errors result in large velocity errors. Particularly for highly dynamic vehicles, velocity errors induced will be more significant. In an UTC integration system, the velocity solutions of the INS are fed back to numerically-controlled oscillator (NCO) command generators. Since velocity errors increase, the local replica carrier frequency will deviate from the true received carrier frequency. Therefore, large MIMU measurement errors will result in large jumps in frequency errors.

Large frequency tracking errors will cause the attenuation of coherent integration values. Further, they cause a sudden decrease in the estimated carrier-to-noise ratio (*C/N*_0_) and an increase in the code tracking error. In highly dynamic environments, if the large frequency tracking error cannot be mitigated in time, it may even result in loss of the tracking lock.

Several previous methods were developed to cope with large frequency offsets. When the oscillator frequency jumps significantly, the apparent *C*/*N*_0_ will suddenly drop for all tracking channels [9]. For this reason, the estimated *C*/*N*_0_ is used as an indicator to detect large frequency errors in [9]. The optimal frequency offset compensation is obtained through a search algorithm. This method is very effective for the large frequency error caused by the crystal oscillator. However, the frequency error caused by MIMU measurement errors depends on the line-of-sight direction of the satellite being tracked. The estimated *C*/*N*_0_ of each tracking channel may not drop suddenly at the same time. For frequency errors induced by MIMU errors, the effect of this technique based on *C*/*N*_0_ is limited.

In recent years, in challenging applications, the GNSS open-loop receiver architecture has gained much attention. A quasi-open loop receiver architecture was recently introduced to estimate the time varying carrier frequency of GNSS signals [14]. An open-loop approach combining batch and sequential signal processing has been demonstrated to improve the tracking sensitivity of a global positioning system (GPS) receiver, and it was successfully applied in the INS/GPS deep integration system [15]. This advanced open-loop receiver architecture applies batch correlation to obtain a coarse signal parameter estimation, while sequential processing is used to perform a fine signal zoom. The main drawback of the open-loop receiver architecture is the lack of computational efficiency. However, the coarse/fine tracking method provides a new perspective for us to solve the problem of large jumps in frequency errors.

We present an enhanced MIMU/GNSS UTC integration system to mitigate performance degradation caused by abrupt changes in frequency tracking errors. The GPS L1 C/A code signal is used as a case study. The difference between the new architecture and conventional systems is mainly reflected in the following ways. First, each tracking channel of the enhanced UTC (EUTC) architecture contains a module for large frequency tracking error detection. It monitors the frequency tracking error by comparing the frequency discriminator outputs and the preset thresholds. Second, a serial refined frequency processor based on retuned frequency is introduced in each tracking channel. The main function of the refined frequency processor is to fix coherent integration results and prevent the tracking loop from being contaminated by large frequency errors. Finally, the channel prefilter based on the classical Kalman filter (CKF) is replaced by an adaptive prefilter with multiple fading factors, which enhances the ability to track large frequency errors.

## 2. Conventional MIMU/GNSS UTC Integration Architecture

The receiver radio frequency (RF) front end converts the analog RF signal into a digital intermediate frequency (DIF) signal, which is input to the baseband signal processing module. The outputs of correlators are accumulated during the predetection integration interval to obtain coherent integration values. The coherent integration values can be expressed as

(1) $$\begin{array}{l} I_{\alpha }\left(n\right)=AD\left(n\right)R\left(\tau_{\alpha }\right)\mathrm{sinc}\left(\pi f_{e}T_{coh}\right)\cos\left(\phi_{e}\right)+w_{I\alpha ,n}\\ Q_{\alpha }\left(n\right)=AD\left(n\right)R\left(\tau_{\alpha }\right)\mathrm{sinc}\left(\pi f_{e}T_{coh}\right)\sin\left(\phi_{e}\right)+w_{Q\alpha ,n} \end{array}$$

where I and Q are the in-phase and quadrature coherent integration values, respectively; α=E,P,L, where *E*, *P*, and *L* represent the early, prompt, and late correlators, respectively; *A* is the amplitude; R(⋅) is the code correlation function; $\tau_{\alpha }$ is the code phase offset; sinc(x)=sin(x)/x is known as the frequency attenuation factor; $T_{coh}$ is the coherent integration time; $f_{e}$ is the carrier frequency error; $\phi_{e}$ is the phase error; and $w_{I\alpha ,n}$ and $w_{Q\alpha ,n}$ denote zero-mean noise terms with standard deviation $\sigma_{n}$.

For UTC navigation systems, the processing methods of the coherent integration values are divided into two categories. The first method is to directly send *I* and *Q* signals to the integration Kalman filter as filter measurements [16,17]. The main problems of this scheme are the high complexity and the high processor load. The second solution is to configure a prefilter for each tracking channel. The code phase and carrier frequency errors of the prefilter output are converted into pseudo-range (PR) and pseudo-range rate (PRR) residuals, which are used as integration filter measurements [7,18,19].

**Figure 1:**
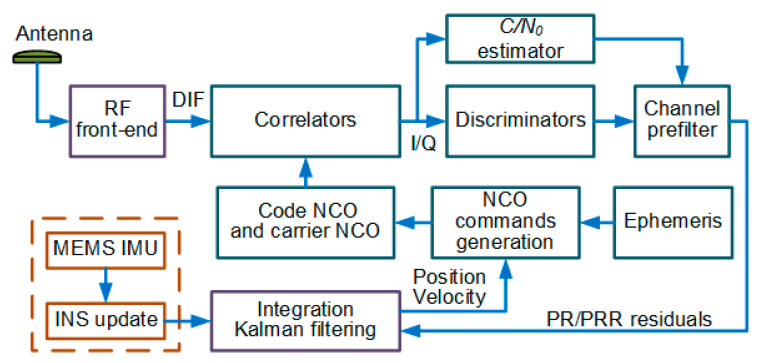
A typical MIMU/GNSS UTC navigation architecture with channel prefilters.

The second scheme is adopted in this paper. However, it is worth noting that the large frequency error mitigation method that we propose is not limited by UTC architectures. Figure 1 depicts a typical embodiment of MIMU/GNSS UTC integration architectures. In the rest of the paper, this architecture is called the conventional UTC (CUTC) architecture.

### 2.1. Channel Prefilter Model

The channel prefilters are utilized to generate PR and PRR measurements as illustrated in Figure 1. In harsh environments, the carrier phase tracking is more vulnerable than the frequency tracking. For this reason, states of the prefilter include code phase error $\tau_{p}$, carrier frequency error $f_{e}$, and carrier frequency rate δa, excluding carrier phase error.

The discrete-time system model of the prefilter is

(2) $$x_{k}=\Phi_{k/k-1}x_{k-1}+\eta_{k-1}$$

where $x={\left[\begin{array}{ccc} \tau_{p} & f_{e} & \delta a \end{array}\right]}^{T}$ is the state vector, and η is the system noise vector.

The state one-step transition matrix is

(3) $$\Phi_{k/k-1}=\left[\begin{array}{ccc} 1 & \beta T & \beta T^{2}/2\\ 0 & 1 & T\\ 0 & 0 & 1 \end{array}\right]$$

where T is the update period, $\beta =f_{co}/f_{ca}$ is a scale factor, $f_{ca}$ is the nominal carrier frequency, and $f_{co}$ is the pseudo-random noise (PRN) code chip rate. For the GPS L1 C/A code, β is equal to 1/1540.

The prefilter measurement model is modeled as

(4) $$z_{k}=Hx_{k}+v_{k}$$

where $z_{k}={\left[\begin{array}{cc} z_{\tau } & z_{f} \end{array}\right]}^{T}$ is the measurement vector, and $v_{k}$ is the measurement noise vector. The variables $z_{\tau }$ and $z_{f}$ denote the outputs of the code discriminator and carrier frequency discriminator, respectively.

The measurement matrix is defined as

(5) $$H=\left[\begin{array}{ccc} 1 & -\beta T/2 & \beta T^{2}/6\\ 0 & 1 & -T/2 \end{array}\right].$$

A normalized non-coherent early-minus-late envelope code discriminator [20] is applied to compute code tracking errors.

(6) $$z_{\tau }=\frac{1}{2}\frac{E-L}{E+L}$$

where *E* and *L* are the autocorrelation amplitudes of the early and late correlators, respectively, given by

(7) $$\begin{array}{l} E=\sqrt{I_{E}^{2}\left(n\right)+Q_{E}^{2}\left(n\right)}\\ L=\sqrt{I_{L}^{2}\left(n\right)+Q_{L}^{2}\left(n\right)} \end{array}.$$

A four-quadrant arctangent frequency discriminator [20] is used in this paper, given by

(8) $$z_{f}=\frac{\arctan2\left(P_{cross},P_{dot}\right)}{\pi T_{coh}}.$$

$P_{cross}$ and $P_{dot}$ in (8) can be expressed as follows:

(9) $$\begin{array}{l} P_{cross}=I_{p,1}Q_{p,2}-Q_{p,1}I_{p,2}\\ P_{dot}=I_{p,1}I_{p,2}+Q_{p,1}Q_{p,2} \end{array}$$

where $I_{p,1}$ and $Q_{p,1}$ denote the coherent integration values of the first half of the coherent integration period, and $I_{p,2}$ and $Q_{p,2}$ denote the coherent integration values of the second half of the coherent integration period.

The code discriminator variance $\sigma_{\tau }^{2}$ and frequency discriminator variance $\sigma_{f}^{2}$ can be approximated as follows [21,22]:

(10) $$\begin{array}{l} \sigma_{\tau }^{2}\approx \frac{1}{\left(c/n_{0}\right)T_{coh}}\left(\frac{1}{2}+\frac{1}{2\left(c/n_{0}\right)T_{coh}}\right)\\ \sigma_{f}^{2}\approx \frac{1}{4\pi^{2}\left(c/n_{0}\right){\left(T_{coh}/2\right)}^{3}}\left(1+\frac{1}{\left(c/n_{0}\right)T_{coh}/2}\right) \end{array}$$

where $c/n_{0}$ is the carrier-to-noise ratio in Hz.

### 2.2. Integration Kalman Filter

The integration Kalman filter estimates INS navigation errors and MIMU errors. The following 17-dimensional error state vector is adopted in this study:

(11) $$X={\left[\begin{array}{ccccccc} \phi^{T} & {\left(\delta v^{n}\right)}^{T} & {\left(\delta p\right)}^{T} & {\left(\varepsilon_{r}^{b}\right)}^{T} & {\left(\nabla_{r}^{b}\right)}^{T} & \delta t_{u} & \delta f_{u} \end{array}\right]}^{T}$$

where ϕ represents the attitude misalignment angles, $\delta v^{n}$ represents the velocity errors, δp is the position (latitude, longitude and altitude) errors, $\varepsilon_{r}^{b}$ and $\nabla_{r}^{b}$ are the first-order Markov process errors of gyroscopes and accelerometers, respectively, $\delta t_{u}$ is the receiver clock error, and $\delta f_{u}$ is the clock drift error.

The system equation of the integration Kalman filter usually comes from the error propagation equation of the INS. The complete form is a complex set of differential equations, which can be found in [1,23].

Equation (12) converts the state estimations of the prefilter into the PR residual and the PRR residual. The residuals of all available channels constitute the measurement vector of the integration filter.

(12) $$\{\begin{array}{c} \Delta \rho =\frac{{\hat{\tau }}_{p}\times c}{f_{co}}\\ \Delta \dot{\rho }=\frac{{\hat{f}}_{e}\times c}{f_{ca}} \end{array}$$

where Δρ is the PR residual, $\Delta \dot{\rho }$ is the PRR residual, ${\hat{\tau }}_{p}$ is the estimated code phase error, ${\hat{f}}_{e}$ is the estimated carrier frequency error, and *c* is the speed of light.

The measurement equation of the UTC Kalman filter is the same as the measurement equation of the standard tight integration system, and for the specific form, one can refer to [24]. In the UTC navigation system, the corrected inertial navigation solutions are applied to generate the carrier and code NCO control commands. A detailed treatment of NCO commands may be found in [24,25].

## 3. Quantitative Analyses of Disruptive Effects of Large Frequency Errors

According to Equation (1), when the tracking channel exhibits a large frequency tracking error, the sinc(⋅) term will cause a large attenuation of the signal power. The signal power attenuation will cause the *C*/*N*_0_ estimation errors and code tracking estimation errors to increase. In this section, we explore the disruptive effects of large frequency tracking errors via theoretical analyses and Monte Carlo simulations.

### 3.1. Effects on the C/N_0_ Estimation

In order to avoid the loss of signal energy induced by the code phase offset, the *C*/*N*_0_ is usually estimated using the coherent integration values from prompt correlators [20]. When a code loop is in a stable tracking state, the code phase tracking error is nominal. The autocorrelation function corresponding to the prompt code phase error $R\left(\tau_{p}\right)$ is equal to one. When the coherent integration time does not cross data bits, the data bit is constant, and the influence on the coherent integration can be omitted. For these reasons, the coherent integration values of the prompt correlators can be simplified as

(13) $$\begin{array}{l} I_{p}\left(n\right)=A\mathrm{sinc}\left(\pi f_{e}T_{coh}\right)\cos\left(\phi_{e}\right)+w_{Ip,n}\\ Q_{p}\left(n\right)=A\mathrm{sinc}\left(\pi f_{e}T_{coh}\right)\sin\left(\phi_{e}\right)+w_{Qp,n} \end{array}.$$

In fact, in Equation (13), the attenuation of the signal amplitude caused by the frequency error $f_{e}$ is very difficult to separate from variation in the signal amplitude *A* [26]. Accordingly, the sinc(⋅) term will cause the estimated *C*/*N*_0_ to decrease significantly in the case of a large frequency error. It should be pointed out that the true *C*/*N*_0_ is not related to $f_{e}$. The frequency error affects the estimated *C*/*N*_0_ from coherent integration values.

In practice, GNSS receivers measure *C*/*N*_0_ by means of various *C*/*N*_0_ estimation algorithms [27,28]. In the GNSS context, the narrow-to-wideband power ratio (NWPR) method is usually used as a standard estimator. Therefore, we used the NWPR method as an example to analyze the effects of large frequency tracking errors on the *C*/*N*_0_ estimation results.

The narrow-to-wide power ratio is expressed as

(14) $$P_{n/w}=\frac{P_{n}}{P_{w}}$$

where $P_{n}$ is the narrowband power, and $P_{w}$ is the wideband power.

When the frequency error is negligible, the expectation of the narrow-to-wide power ratio is defined as [27]

(15) $$E\left(P_{n/w}\right)\approx \frac{M\left(c/n_{0}\tau +1\right)}{M+c/n_{0}\tau }$$

where $\tau =MT_{coh}$ and *M* is the length of the data bit in milliseconds. For the GPS L1 C/A code, M=20 is commonly adopted.

When the frequency error cannot be ignored, the expectation of the narrow-to-wide power ratio can be expressed as follows (see Appendix A):

(16) $$E\left({\tilde{P}}_{n/w}\right)\approx \frac{M\left(c/n_{0}\tau {\mathrm{sinc}}^{2}\left(\pi f_{e}\tau \right)+1\right)}{M+c/n_{0}\tau {\mathrm{sinc}}^{2}\left(\pi f_{e}T_{coh}\right)}.$$

In the NWPR method, *N* power ratios are usually averaged to reduce the effects of noise on the statistical results. The average value is given by

(17) $$\mu_{P}=\frac{1}{N}\sum_{k=1}^{N}P_{n/w}\left(k\right).$$

Suppose that the number of power ratios that can ignore the effect of frequency errors is $N_{1}$, and the number of power ratios that are impaired by large frequency errors is $N_{2}$. Then $N=N_{1}+N_{2}$. The expectation of $\mu_{p}$ is

(18) $$E\left(\mu_{P}\right)=\frac{N_{1}}{N}E\left(P_{n/w}\right)+\frac{N_{2}}{N}E\left({\tilde{P}}_{n/w}\right).$$

In the presence of large frequency errors, the estimated *C*/*N*_0_ is modeled as

(19) $$\tilde{c}/{\tilde{n}}_{0}=\frac{1}{T_{coh}}\frac{E\left(\mu_{P}\right)-1}{M-E\left(\mu_{P}\right)}.$$

If there is no large frequency error, the estimated *C*/*N*_0_ can be expressed as

(20) $$c/n_{0}=\frac{1}{T_{coh}}\frac{E\left(P_{n/w}\right)-1}{M-E\left(P_{n/w}\right)}.$$

The theoretical *C*/*N*_0_ attenuation due to large frequency tracking errors is obtained as

(21) $$G_{nwpr}=10\log_{10}\left(\frac{\tilde{c}/{\tilde{n}}_{0}}{c/n_{0}}\right).$$

Theoretical analysis and Monte Carlo (MC) simulation were used to quantify the influence of frequency errors on the *C*/*N*_0_ estimation. The theoretical *C*/*N*_0_ attenuation was obtained from Equations (19)–(21). *M* was set to 20. *T_coh_* and *τ* were 1 ms and 20 ms, respectively. In the Monte Carlo simulation, the true *C*/*N*_0_ was set to 40 dB-Hz. Again, *N* was set to 50, that is, the power ratio was averaged over a time interval of 1 s. The estimated *C*/*N*_0_ and the true *C*/*N*_0_ were substituted into Equation (21) to obtain the *C*/*N*_0_ attenuation.

**Figure 2:**
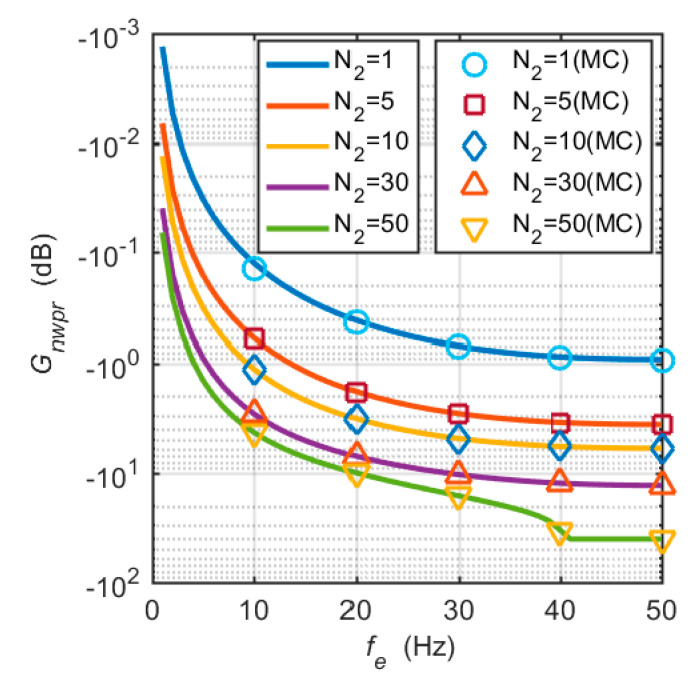
Estimated *C*/*N*_0_ attenuation caused by frequency tracking errors. The solid lines represent the theoretical analysis results, and Monte Carlo simulation results are represented by discrete points.

Figure 2 shows the theoretical *C/N*_0_ attenuation and the 1000 Monte Carlo simulation results. The two sets of results are relatively consistent. For the case of $N_{2}=1$, the attenuation of the estimated *C*/*N*_0_ caused by large frequency errors is less than 1 dB, and the effect of frequency errors is negligible. However, when $N_{2}$ is greater than 5 and frequency errors are greater than 20 Hz, the effect of frequency errors on the estimated *C*/*N*_0_ is very significant. When $N_{2}=50$ and $f_{e}>30$ Hz, the ${\mathrm{sinc}}^{2}\left(\cdot \right)$ term in the numerator of Equation (16) quickly decays to near zero. The estimated *C*/*N*_0_ and *C*/*N*_0_ attenuation also drop sharply. Therefore, there is a jump in the *C*/*N*_0_ attenuation curve of $N_{2}=50$ at $f_{e}=40$ Hz. In addition, we limited the minimum value of *C*/*N*_0_ to 0 dB-Hz, that is, the maximum attenuation of *C*/*N*_0_ is –40 dB, as seen in Figure 2.

### 3.2. Effects on the Code Tracking Error Estimation

When a large frequency tracking error occurs, if the coherent integration time is limited so as to not exceed the data bit interval, Equation (1) can be rewritten as follows:

(22) $$\begin{array}{l} I_{\alpha }\left(n\right)=AR\left(\tau_{\alpha }\right)\mathrm{sinc}\left(\pi f_{1}n_{1}T_{1}\right)\cos\left(\phi_{1}\right)+AR\left(\tau_{\alpha }\right)\mathrm{sinc}\left(\pi f_{2}n_{2}T_{1}\right)\cos\left(\phi_{2}\right)+w_{I\alpha ,n}\\ Q_{\alpha }\left(n\right)=AR\left(\tau_{\alpha }\right)\sin c\left(\pi f_{1}n_{1}T_{1}\right)\sin\left(\phi_{1}\right)+AR\left(\tau_{\alpha }\right)\mathrm{sinc}\left(\pi f_{2}n_{2}T_{1}\right)\sin\left(\phi_{2}\right)+w_{Q\alpha ,n} \end{array}$$

where $f_{1}$ is the trivial frequency error; $f_{2}$ is the large frequency error; $T_{1}$ denotes the pseudo-code period, where for the GPS L1 C/A, $T_{1}$ is 1 ms; $n_{1}$ and $n_{2}$ are the number of pseudocode periods corresponding to $f_{1}$ and $f_{2}$, respectively; and $\phi_{1}$ and $\phi_{2}$ are phase errors. In Equation (22), for simplicity, it is assumed that large frequency errors are aligned with the pseudocode periods. Phase errors can be expressed as

(23) $$\begin{array}{l} \phi_{1}=\pi f_{1}n_{1}T_{1}+\theta_{e}\\ \phi_{2}=\pi f_{2}n_{2}T_{1}+2\pi f_{1}n_{1}T_{1}+\theta_{e} \end{array}$$

where $\theta_{e}$ is the initial phase error.

The autocorrelation amplitudes *E* and *L* in Equation (7) are attenuated by the sinc(⋅) term in the case of a large frequency error, resulting in an increase in the code discriminator error. Since the probability distributions of *E* and *L* follow the complex Rice distribution, it is difficult to obtain the analytical expression of the effect of frequency error on the code discriminator. The Monte Carlo method was applied to numerical simulation analyses. For the code discriminator, the coherent integration time was set to 20 ms. The time range of large frequency errors was 0–20 ms, that is, in Equation (22), $1\leq n_{2}\leq 20$ The true *C*/*N*_0_ was set to 40 dB-Hz.

**Figure 3:**
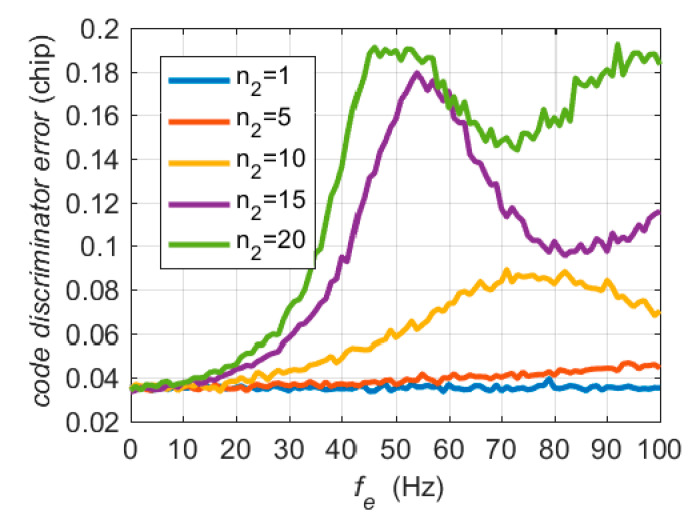
RMS errors of the code discriminator.

Figure 3 shows the root mean square (RMS) errors of the code discriminator in Equation (6) for 1000 Monte Carlo simulations. When the frequency error is close to 0, the RMS errors of the code discriminator is less than 0.04 chips. The maximum code discriminator output error caused by a frequency error is close to 0.2 chips, which is about five times that without a frequency tracking error.

## 4. Proposed MIMU/GNSS UTC Integration Architecture

The results in the previous section indicate that the large frequency tracking error causes the estimated *C*/*N*_0_ to rapidly decrease. Furthermore, the code discriminator estimation error caused by the large frequency error is greater than the error without frequency error. If the large frequency tracking error cannot be eliminated in time, especially in highly dynamic environments, it will seriously impair the performance of the navigation system.

**Figure 4:**
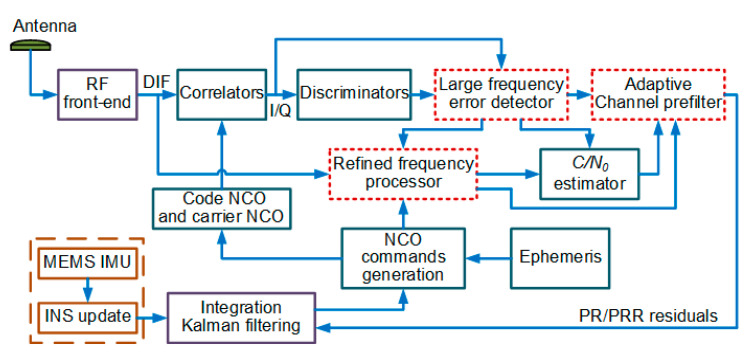
A block diagram of the proposed UTC architecture.

In order to enhance the performance of the UTC navigation system under large frequency errors, it is necessary to improve the CUTC integration architecture shown in Figure 1. In this section, we propose a new UTC integration architecture. Figure 4 summarizes the structure of the proposed MIMU/GNSS UTC architecture.

The architecture is referred to as the enhanced UTC (EUTC) integration in the rest of the paper. A large frequency error detector and a serial refined frequency processor based on retuned frequency are added to each tracking loop to reduce the impact of large frequency tracking errors on the coherent integration results. In addition, an adaptive prefilter is used to replace the conventional channel prefilter to enhance the estimation performance of abrupt frequency errors. Next, we introduce the principles of these three modules in detail.

### 4.1. Large Frequency Error Detector

The limited operating range of the time domain frequency discriminator is an important reason for the degradation of system performance induced by large frequency errors. If the frequency error exceeds the pull-in range, the frequency discriminator outputs an erroneous estimate.

**Figure 5:**
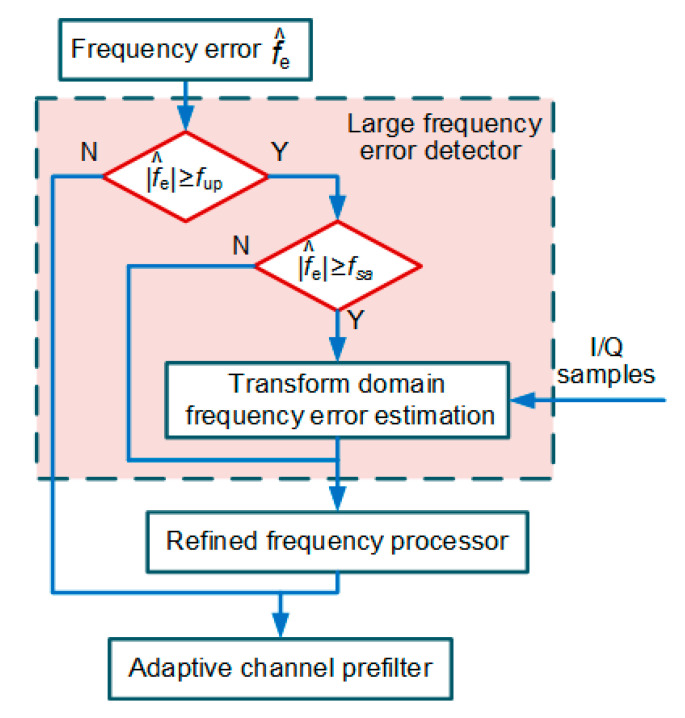
Flow diagram of the large frequency error detector.

In the EUTC system, the frequency error output by the frequency discriminator is first forwarded to the large frequency error detector. The main purpose of the detector is to detect whether the current frequency tracking error is anomalous. Additionally, if the frequency error exceeds the working range of the time domain frequency discriminator, a correct frequency error estimate is provided by the detector. Figure 5 depicts a detailed flow chart of the large frequency error detector.

In the large frequency error detector, the frequency error ${\hat{f}}_{e}$ output from the four-quadrant arctangent frequency discriminator is first compared with the preset upper limit $f_{up}$. In our study, $f_{up}$ is set to 20 Hz. If the absolute value of ${\hat{f}}_{e}$ is less than the threshold $f_{up}$, the current frequency tracking is normal, and the frequency error ${\hat{f}}_{e}$ is passed to the channel prefilter. Moreover, the original coherent integration results are directly inputted to the NWPR *C*/*N*_0_ estimator. Otherwise, it means that the current epoch is affected by a large frequency tracking error.

Subsequently, it is further determined whether the frequency error exceeds the range of the discriminator. If the absolute value of ${\hat{f}}_{e}$ is greater than or equal to the discriminator threshold $f_{sa}$, the discriminator output is unreliable. In our study, $f_{sa}$ is set to the single-sided frequency pull-in range multiplied by 0.8. For example, with a 10 ms predetection integration time, the single-sided frequency pull-in range is 50 Hz, and $f_{sa}$ is equal to 40 Hz. In order to obtain the correct frequency error, a transform domain frequency discriminator [15,29] with a larger working range is applied to reprocess the coherent integration results. The new frequency error estimate is input to the refined frequency processor. In contrast, the frequency error that does not exceed the pull-in range of the discriminator is directly inputted to the refined frequency processor.

### 4.2. Refined Frequency Processor

**Figure 6:**
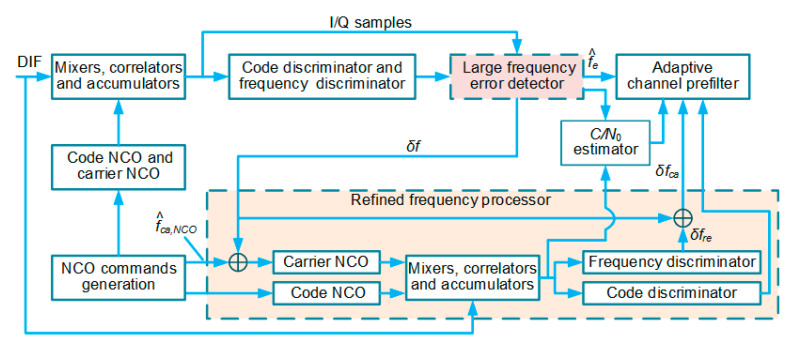
A block diagram of the refined frequency processor.

Another reason for the system performance degradation induced by large frequency errors is the loss of consistency between the replica carrier signal and the incoming signal. We introduce refined frequency processors to overcome this problem. The generalized structure of the refined frequency processor is shown in Figure 6.

The refined frequency processor is the core module of the EUTC integration system. Its main function is to fix coherent integration results. The operation of the refined frequency processor can be divided into three steps.

First, according to the frequency error output by the large frequency error detector, the retuned replica carrier frequency is obtained as

(24) $$f_{ca,re}={\hat{f}}_{ca,NCO}+\delta f$$

where δf denotes the output of the large frequency error detector.

Second, the mixing and correlation operations of the current epoch are performed, again based on the retuned carrier frequency. Since the code frequency is usually much smaller than the carrier frequency, the code Doppler frequency shift is also much smaller than the carrier Doppler frequency shift. If the coherent integration time does not exceed 20 ms, the code phase offset caused by a large frequency error is not significant. Therefore, in most cases, we do not need to correct the code Doppler shift and can continue to use the code NCO command of the current epoch in the refined frequency processor. The correlators output a new set of coherent integration values.

Thirdly, the code phase error and frequency error are estimated based on new coherent integration values. At this point, the frequency error is nominal. A four-quadrant arctangent frequency discriminator is employed in the refined frequency processor. Moreover, the new coherent integration results are inputted to the *C*/*N*_0_ estimator to obtain a more accurate *C*/*N*_0_ estimate. The carrier frequency tracking error of the current epoch can be expressed as

(25) $$\delta f_{ca}=\delta f+\delta f_{re}$$

where $\delta f_{re}$ is the frequency discriminator output in the refined frequency processor.

Note that the refined frequency processor does not significantly reduce the operating efficiency of a GNSS receiver. In most operating environments, carrier frequency tracking errors do not exceed the threshold. Therefore, it is not necessary to call the refined frequency processor frequently. In addition, since there is no need to adjust the code frequency, the sample values of the three kinds of replica code sequences (early, prompt, and late) can be saved during the first correlation operation. In this way, in the refined frequency processor, the code NCOs generating replica code sequences can be omitted. For these reasons, the refined frequency processor will not significantly increase the computational burden of the receiver.

### 4.3. Adaptive Prefilter with Multiple Fading Factors

The conventional channel prefilter depicted in Section 2.1 usually uses a CKF. A fatal drawback of the CKF is its poor performance in estimating mutation states. For the CKF in the steady state, the state covariance matrix of the filter tends to a fixed value, causing the gain matrix of the Kalman filter to tend to zero. In other words, for a steady-state CKF, the measurement correction is weakened. If a large frequency tracking error suddenly occurs, the estimated frequency error of a conventional prefilter will deviate from its true value. In this case, the CKF exhibits a serious model mismatch. An adaptive Kalman filter is an effective method to counteract the problem [30]. An adaptive Kalman filter with multiple fading factors is proposed to construct an adaptive channel prefilter.

According to the basic theory of Kalman filtering, if there is no model mismatch, the innovation sequence of a CKF is a white noise sequence. In the process of filter updating, the variance matrix of the innovation sequence can be estimated by the following recursive expression [31]:

(26) $${\hat{V}}_{k}=\left(1-\beta_{k}\right){\hat{V}}_{k-1}+\beta_{k}e_{k}e_{k}^{T}$$

where ${\hat{V}}_{k}$ is the estimate of the variance matrix at time *k*, ${\hat{V}}_{k-1}$ is the variance matrix estimate of the previous epoch, $\beta_{k}$ is the fading weighting factor, and $e_{k}$ is the innovation vector.

The calculation of the fading weighting factor is a recursive process. The update method is expressed as follows [31]:

(27) $$\beta_{k}=\frac{\beta_{k-1}}{\beta_{k-1}+b}$$

where $\beta_{0}=1$ is the initial value, and b is a preset constant with a value range of 0.9–0.999.

${\hat{V}}_{k}$ is approximately equal to the theoretical innovation variance matrix, given by

(28) $${\hat{V}}_{k}\approx H_{k}\left(\Phi_{k/k-1}P_{k-1}\Phi_{k/k-1}^{T}+Q_{k-1}\right)H_{k}^{T}+R_{k}$$

where $H_{k}$ is the measurement matrix, $\Phi_{k/k-1}$ is the state transition matrix, $P_{k-1}$ is the state covariance matrix, $Q_{k-1}$ is the system noise matrix, and $R_{k}$ is the measurement noise matrix.

The channel prefilter contains three state variables. For filters with multiple state variables, if the uncertainty of the model has dissimilar effects on different states, multiple fading factors should be used to adaptively adjust the different state components.

The multiple fading factor matrix $\lambda_{k}$ is defined as

(29) $$\begin{array}{l} \lambda_{k}=\sqrt{c_{k}}\alpha \\ \alpha =\mathrm{diag}\left(\sqrt{\alpha_{1}},\sqrt{\alpha_{2}},\cdots ,\sqrt{\alpha_{n}}\right) \end{array}$$

where $c_{k}$ is the coefficient to be calculated, diag(⋅) denotes a diagonal matrix composed of elements in the parentheses, and $\alpha_{i}\geq 1$ is the fading proportional coefficient corresponding to the *i*-th component.

The coefficient $c_{k}$ is a scalar parameter, expressed as follows:

(30) $$c_{k}=\frac{\mathrm{tr}\left(N_{k}\right)}{\mathrm{tr}\left(M_{k}\right)}$$

where tr(⋅) represents the matrix trace operation. Detailed expressions of $N_{k}$ and $M_{k}$ are provided as follows:

(31) $$\begin{array}{l} N_{k}={\hat{V}}_{k}-H_{k}Q_{k-1}H_{k}^{T}-R_{k}\\ M_{k}=H_{k}\Phi_{k/k-1}\left(\alpha P_{k-1}\alpha \right)\Phi_{k/k-1}^{T}H_{k}^{T} \end{array}.$$

Note that the lower limit of the fading factor is 1. $\lambda_{k}$ is a diagonal matrix. The diagonal element $\lambda_{k}\left(i,i\right)$ is the fading factor corresponding to the *i*-th state component, defined as follows:

(32) $$\lambda_{k}\left(i,i\right)=\max\left(1,\sqrt{c_{k}}\alpha \right).$$

The difference between the adaptive Kalman filter with multiple fading factors and the classic Kalman filter is the time update of the state covariance matrix. The update process of the former is as follows:

(33) $$P_{k/k-1}=\Phi_{k/k-1}\left(\lambda_{k}P_{k-1}\lambda_{k}\right)\Phi_{k/k-1}^{T}+Q_{k-1}.$$

The state-space model of the adaptive channel prefilter is the same as the conventional prefilter. The code phase error is not prone to sudden changes. Therefore, the corresponding fading proportional coefficient α is set to 1.

## 5. Simulation Experiment Results

In this section, simulation experiment results are presented to evaluate the performance of the EUTC integration system in the presence of large frequency errors.

### 5.1. Simulation Setup

The trajectory devised for simulations is a highly dynamic flight profile in the horizontal plane. A GNSS simulator was used to generate GPS L1 C/A RF signals corresponding to the reference trajectory. The RF signals were collected with an intermediate frequency collector. The IF collector converted the simulated RF signals into digital IF signals and stored them on a computer for post-processing.

**Figure 7:**
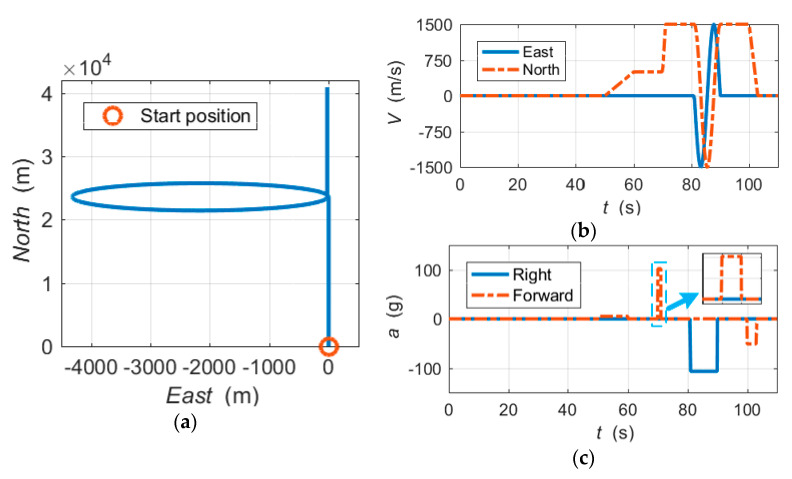
Simulation reference trajectory: (**a**) horizontal trajectory; (**b**) velocity profiles; and (**c**) acceleration profiles.

Figure 7a shows the horizontal trajectory. Figure 7b,c shows the velocity and acceleration profiles of the simulation vehicle, respectively. The typical parameters of the simulation trajectory are as follows:

- Starting position: longitude (108.9°), latitude (34.2°) and altitude (350 m);
- Trajectory duration: 110 s;
- Maximum velocity: 1500 m/s;
- Highly dynamic segment 1: linear acceleration = 100 g, time interval = (70, 71) s;
- Highly dynamic segment 2: centripetal acceleration > 100 g, time interval = (81, 90) s.

Again, the MIMU data was also simulated based on the reference trajectory. The error parameter settings of the MIMU are provided in Table 1.

The MIMU/GNSS UTC integration simulation program takes digital IF signals and the simulated MIMU data as input data. The UTC navigation simulation parameters are summarized in Table 2.

If large frequency errors and large dynamics occur at the same time, the performance of the UTC navigation system will be greatly impaired. This test case is a severe challenge for the integrated navigation system, which can be regarded as a stringent assessment case. For this reason, in the simulation, a large frequency error was set to appear in the linear acceleration section of 100 g. In most applications, if the step change in frequency exceeds 50 Hz, the replica signal will no longer correlate with the incoming signal [9]. Hence, the large frequency error was set to 10 m/s, and the equivalent GPS L1 carrier frequency offset was 52.5 Hz.

In the simulation, the *C*/*N*_0_ of all tracking channels was set to the same value (50 dB-Hz), so the tracking status of each tracking channel was similar. In the following, one of the tracking channels is chosen as an example to show the channel-dependent simulation results. Before the system was switched to the UTC mode, it was necessary to perform the scalar tracking and tight integration. The simulation results of the UTC integration were obtained starting from 40 s.

### 5.2. Results and Analysis

A CUTC integration simulation was performed as a benchmark for comparative analyses. The implementation details of the CUTC architecture are described in Section 2. The simulation parameter configuration is shown in Table 2.

**Figure 8:**
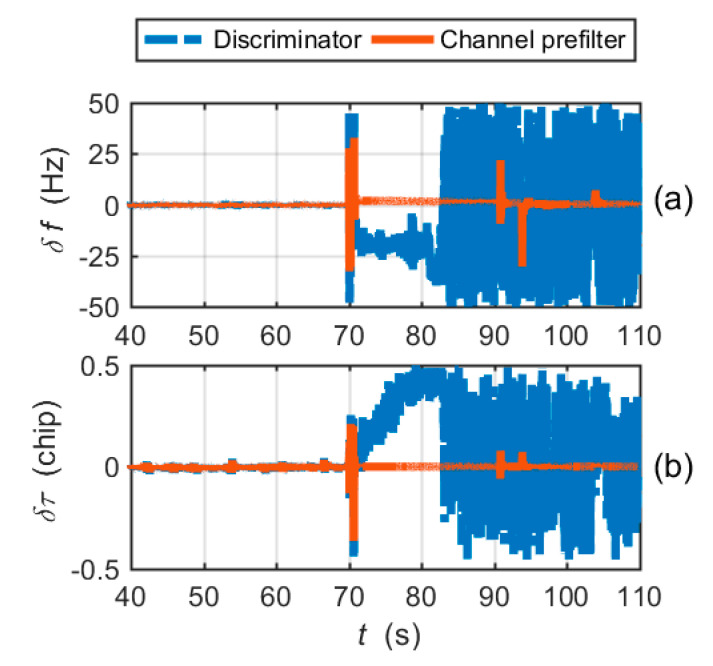
Tracking errors for the CUTC integration simulation: (**a**) carrier frequency tracking errors; (**b**) code phase tracking errors.

Figure 8a shows the carrier frequency tracking errors output by the frequency discriminator and the channel prefilter. The PRN code phase tracking errors are shown in Figure 8b. It is clear that the CUTC architecture cannot mitigate large frequency tracking errors in the tracking loop. The large frequency tracking errors induce the code tracking errors to increase. After 81 s, the highly dynamic circular maneuver causes the carrier frequency tracking and code tracking to lose lock.

**Figure 9:**
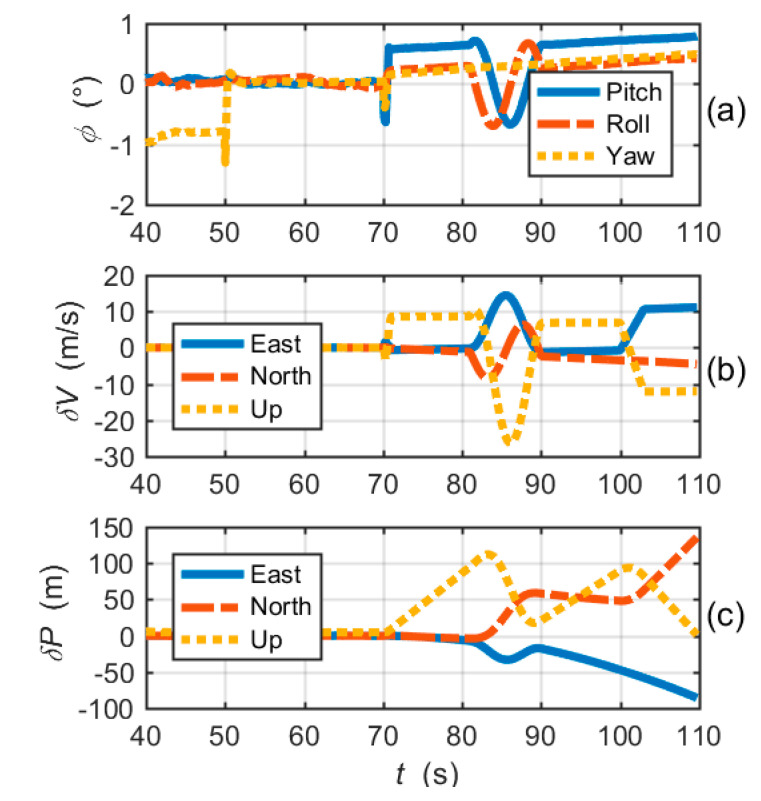
Navigation solution errors of the CUTC integration simulation: (**a**) attitude errors; (**b**) velocity errors; and (**c**) position errors.

Figure 9a–c shows the attitude errors, the velocity errors and the position errors of the CUTC system, respectively. Large frequency errors cause attitude errors and velocity errors to increase rapidly. Position errors gradually diverge with time. In fact, after 81s, due to the loss of lock of tracking loops, integrated navigation errors are essentially inertial navigation errors.

Next, we focused on the simulation analysis of the proposed EUTC system. The simulation of the EUTC integration also used the parameters shown in Table 2.

**Figure 10:**
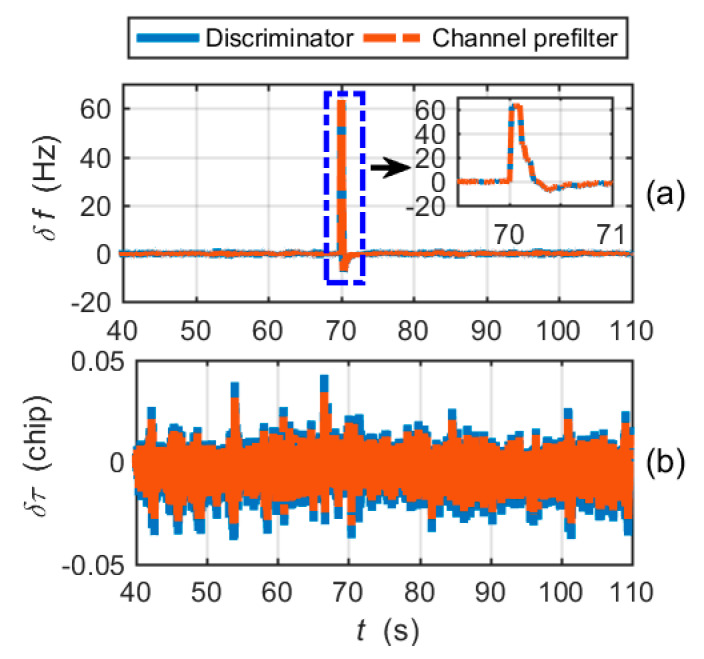
Tracking errors for the EUTC integration simulation: (**a**) carrier frequency tracking errors; (**b**) code phase tracking errors.

Figure 10 shows the frequency and code phase errors of the EUTC navigation system. The simulation results presented demonstrate that the proposed EUTC architecture can eliminate large changes of carrier frequency errors in time. From the magnified view of Figure 10a, we can see that the adaptive prefilter can correctly estimate the frequency error of the abrupt change. When the frequency tracking error changes suddenly, the code tracking loop remains stable.

**Figure 11:**
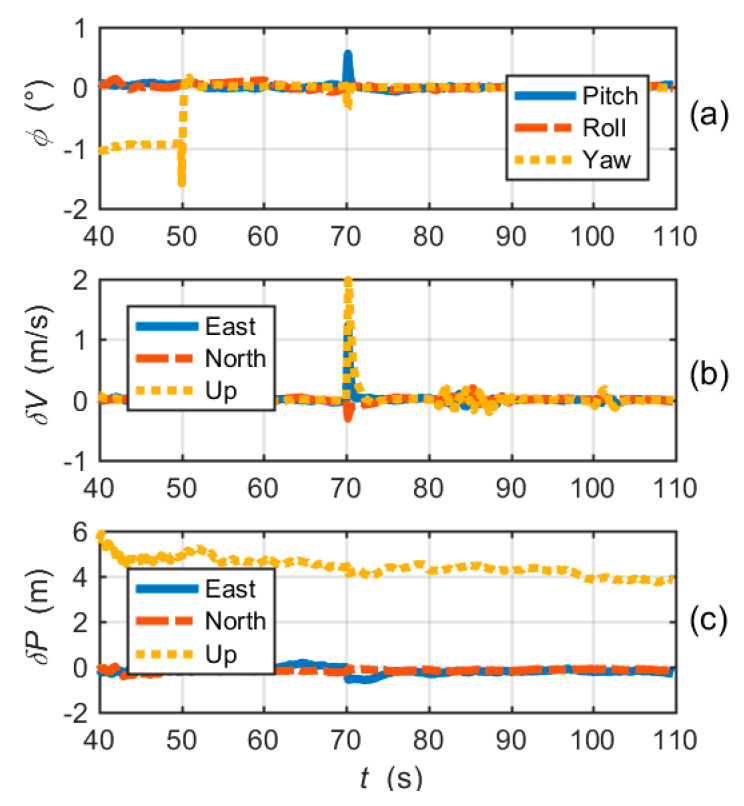
Navigation solution errors of the EUTC integration simulation: (**a**) attitude errors; (**b**) velocity errors; and (**c**) position errors.

Figure 11 shows the navigation errors of the proposed EUTC architecture. At the moment when large frequency errors occur, the pitch angle error and velocity errors (east and up) increase instantaneously. After 70 s, attitude and velocity errors quickly decrease, as shown in the figure. Moreover, Figure 11c shows that there is no significant change in position errors. Hence, the instantaneous increases in attitude and velocity errors have little effect on the overall navigation performance. Comparing Figure 9 and Figure 11, we can conclude that the EUTC architecture can suppress the divergence of navigation errors caused by large frequency errors.

**Figure 12:**
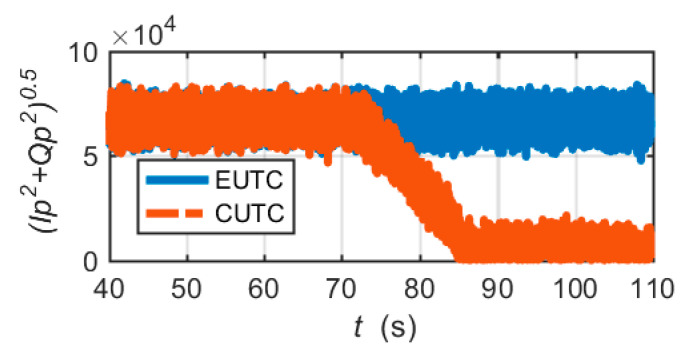
Comparison of 1 ms correlation amplitudes of the prompt correlators.

Figure 12 shows the comparison of the 1 ms correlation amplitudes of the prompt correlators. After 80 s, the correlation amplitude of the CUTC system decays to trivial values, which implies that the tracking loop loses lock. In contrast, the correlation amplitude of the EUTC system is always maintained in the normal range. The EUTC system can maintain continuous operation of the tracking loop when a large frequency error occurs.

**Figure 13:**
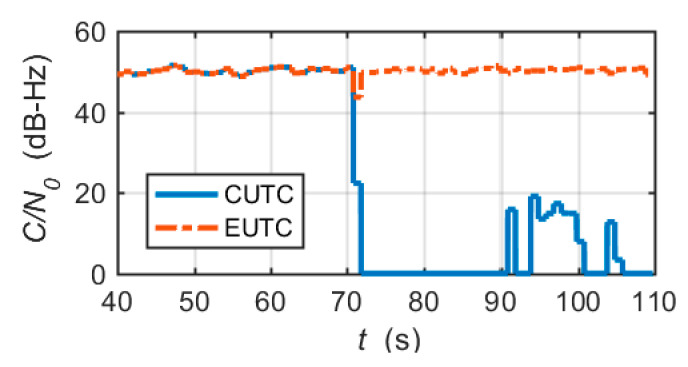
Comparison of *C*/*N*_0_ estimates calculated by the NWPR method.

Figure 13 shows the comparison of the estimated *C*/*N*_0_ of the two integrated architectures. We observe that the estimated *C*/*N*_0_ of the CUTC system rapidly drops to zero in the presence of a large frequency error. The estimated *C/N*_0_ of the EUTC system decreases only slightly in a short time. After 70 s, the estimated *C*/*N*_0_ of the EUTC system recovers quickly. Hence, the EUTC integration can provide continuous and accurate *C*/*N*_0_ estimates.

Finally, a simplified EUTC integration system was simulated to evaluate the performance of the adaptive prefilter. The simplified EUTC integration system comprises large frequency error detectors and refined frequency processors, but uses the same conventional channel prefilters as the CUTC integration.

**Figure 14:**
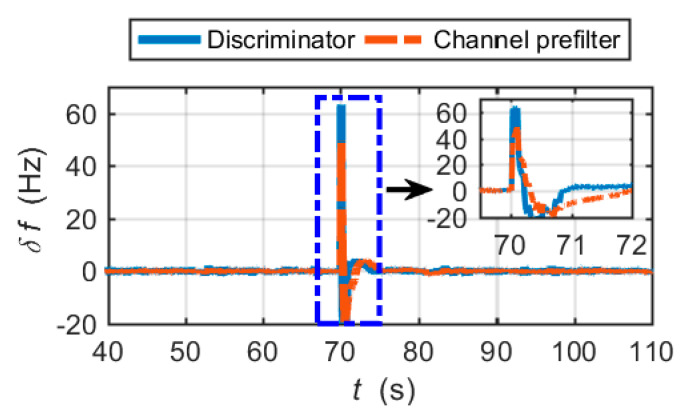
Frequency tracking errors of the simplified EUTC integration.

The frequency tracking errors are shown in Figure 14. The results show that the conventional prefilter cannot track the large changes in frequency errors in time. Referring to Figure 10a, the adaptive prefilter significantly reduces the hysteresis of large frequency error estimation.

**Figure 15:**
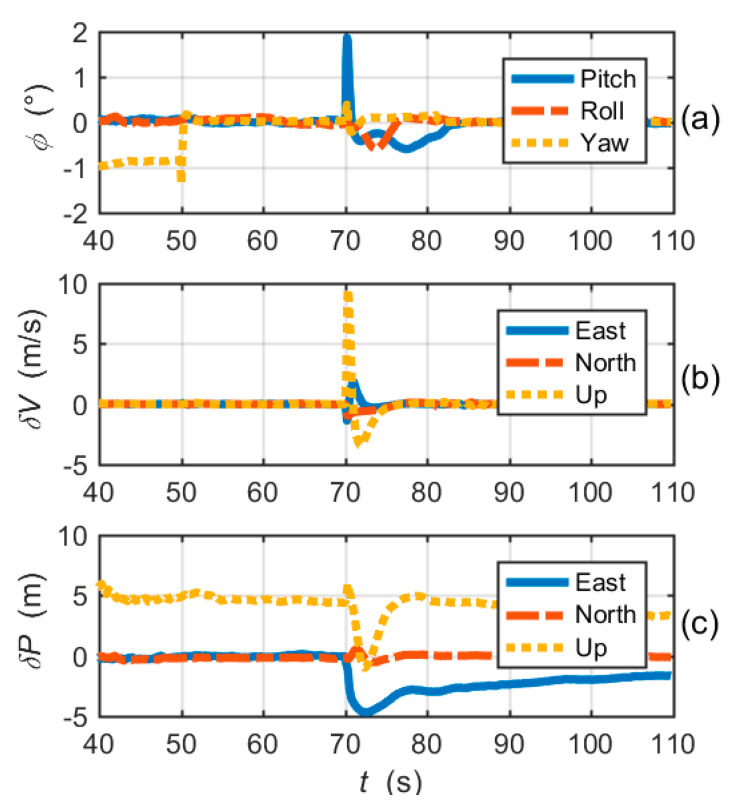
Navigation solution errors of the simplified EUTC integration simulation: (**a**) attitude errors; (**b**) velocity errors; and (**c**) position errors.

The attitude, velocity and position errors of the simplified EUTC integration are shown in Figure 15. Evidently, the convergences of navigation errors are significantly slower than the EUTC integration system using adaptive prefilters. The navigation errors are obviously larger than the navigation errors of the EUTC integration shown in Figure 11. In other words, the adaptive prefilter can help to reduce navigation errors caused by large frequency errors.

## 6. Conclusions

An enhanced MIMU/GNSS UTC integration architecture was proposed for mitigating abrupt changes in the frequency errors. Compared with a conventional UTC integration system, the novel architecture introduces a large frequency error detector and a refined frequency processor on each tracking channel. In the improved architecture, the conventional channel prefilter is substituted by an adaptive prefilter. Using GPS L1 C/A signals as a case study, computer simulation results demonstrate that the enhanced UTC integration system can significantly improve the signal tracking performance in the presence of large frequency errors. The divergence of navigation errors caused by large frequency errors in a highly dynamic environment is avoided. The estimated *C*/*N*_0_ output by the enhanced UTC integration is hardly affected by large frequency errors. Again, with the aid of the adaptive prefilter, the maximum velocity error does not exceed 2 m/s. Position errors are also significantly smaller than the counterpart without the adaptive prefilter.

## Figures and Tables

**Table 1:** MIMU simulation error parameters.

Items	Gyroscope	Accelerometer
Bias	30°/h	500 μg
Random walk	0.3°/sqrt(h) ^1^	50 μg/sqrt(Hz) ^1^
Time-dependent bias	1°/h	50 μg
Correlation time	300 s	300 s
Scale factor error	500 ppm	200 ppm

^1^ sqrt(·) denotes the square root operation.

**Table 2:** UTC navigation simulation parameters.

Types	Parameters	Values
Initial errors	Initial attitude errors	(0.1; 0.1; 2)°
	Initial velocity errors	(0.1; 0.1; 0.1) m/s
	Initial position errors	(3; 3; 5) m
Prefilter process noise variances	Code phase	8 × 10^−4^ (code chip) ^2^
	Carrier frequency	0.1 (Hz) ^2^
	Carrier frequency rate	5 (Hz/s) ^2^
Update frequency	Integration filter	10 Hz ^1^
	Prefilter	50 Hz ^2^
	Code NCO	50 Hz
	Carrier NCO	1000 Hz

^1^ Measurement update frequency. ^2^ Time update frequency is the same as the measurement update frequency.

## Appendix A

The amplitude *A* in Equation (13) can be expressed as

(A1) $$A=\sigma_{n}\sqrt{2\left(c/n_{0}\right)T_{coh}}$$

where $\sigma_{n}$ is the standard deviation of noise terms in (13), $c/n_{0}$ is the carrier-to-noise ratio in Hz, and $T_{coh}$ is the coherent integration time.

Substituting Equations (13) and (A1) into Equation (7) in [27], it is possible to restate the narrowband power as

(A2) $$\begin{array}{l} P_{n}={\left(\sum_{n=1}^{M}I_{p}\left(n\right)\right)}^{2}+{\left(\sum_{n=1}^{M}Q_{p}\left(n\right)\right)}^{2}\\ =M^{2}A^{2}{\mathrm{sinc}}^{2}\left(\pi f_{e}T_{coh}\right)+{\left(\sum_{n=1}^{M}w_{Ip,n}\right)}^{2}+{\left(\sum_{n=1}^{M}w_{Qp,n}\right)}^{2}\\ +2MA\mathrm{sinc}\left(\pi f_{e}T_{coh}\right)\cos\left(\phi_{e}\right)\sum_{n=1}^{M}w_{Ip,n}+2MA\mathrm{sinc}\left(\pi f_{e}T_{coh}\right)\sin\left(\phi_{e}\right)\sum_{n=1}^{M}w_{Qp,n} \end{array}$$

Similarly, the wideband power can be expressed as

(A3) $$\begin{array}{l} P_{w}=\sum_{n=1}^{M}\left(I_{p}^{2}\left(n\right)+Q_{p}^{2}\left(n\right)\right)\\ =MA^{2}{\mathrm{sinc}}^{2}\left(\pi f_{e}T_{coh}\right)+\sum_{n=1}^{M}w_{Ip,n}^{2}+\sum_{n=1}^{M}w_{Qp,n}^{2}\\ +2A\mathrm{sinc}\left(\pi f_{e}T_{coh}\right)\cos\left(\phi_{e}\right)\sum_{n=1}^{M}w_{Ip,n}+2A\mathrm{sinc}\left(\pi f_{e}T_{coh}\right)\sin\left(\phi_{e}\right)\sum_{n=1}^{M}w_{Qp,n} \end{array}$$

In order to calculate the mean and variance of $P_{n}$ and $P_{w}$, we introduce the following variables:

(A4) $$\{\begin{array}{c} X_{I}=\sum_{n=1}^{M}w_{Ip,n}\\ X_{Q}=\sum_{n=1}^{M}w_{Qp,n} \end{array}$$

where $X_{I}$ and $X_{Q}$ are normal random variables with mean 0 and variance $M\sigma_{n}^{2}$.

The expectation of $X_{I}^{2}$ and $X_{Q}^{2}$ is

(A5) $$E\left(X_{I}^{2}\right)=E\left(X_{Q}^{2}\right)=M\sigma_{n}^{2}.$$

In addition, the following variables are defined:

(A6) $$\{\begin{array}{c} Y_{I}=\sum_{n=1}^{M}w_{Ip,n}^{2}\\ Y_{Q}=\sum_{n=1}^{M}w_{Qp,n}^{2} \end{array}.$$

The expectation of $Y_{I}$ and $Y_{Q}$ is

(A7) $$E\left(Y_{I}\right)=E\left(Y_{Q}\right)=M\sigma_{n}^{2}.$$

In addition,

(A8) $$\begin{array}{l} E\left(w_{Ip,n}^{3}\right)=E\left(w_{Qp,n}^{3}\right)=0\\ E\left(w_{Ip,n}^{4}\right)=E\left(w_{Qp,n}^{4}\right)=3\sigma_{n}^{4} \end{array}.$$

Based on Equations (A4)–(A8), the mean and variance of $P_{n}$ are derived as follows:

(A9) $$\begin{array}{l} E\left(P_{n}\right)=2M^{2}\sigma_{n}^{2}\left(c/n_{0}\right)T_{coh}{\mathrm{sinc}}^{2}\left(\pi f_{e}T_{coh}\right)+2M\sigma_{n}^{2}\\ \sigma^{2}\left(P_{n}\right)=4M^{2}\sigma_{n}^{4}\left[2M\left(c/n_{0}\right)T_{coh}{\mathrm{sinc}}^{2}\left(\pi f_{e}T_{coh}\right)+1\right] \end{array}.$$

Similarly, the mean and variance of $P_{w}$ are obtained as follows:

(A10) $$\begin{array}{l} E\left(P_{w}\right)=2M\sigma_{n}^{2}\left(c/n_{0}\right)T_{coh}{\mathrm{sinc}}^{2}\left(\pi f_{e}T_{coh}\right)+2M\sigma_{n}^{2}\\ \sigma^{2}\left(P_{w}\right)=8M\sigma_{n}^{4}\left(c/n_{0}\right)T_{coh}{\mathrm{sinc}}^{2}\left(\pi f_{e}T_{coh}\right)+4M\sigma_{n}^{4} \end{array}.$$

The covariance of $P_{n}$ and $P_{w}$ is

(A11) $$\mathrm{cov}\left(P_{n},P_{w}\right)=4M\sigma_{n}^{4}\left[2M\left(c/n_{0}\right)T_{coh}{\mathrm{sinc}}^{2}\left(\pi f_{e}T_{coh}\right)+1\right].$$

Using Equation (12) in [27], the expectation of the narrow-to-wide power ratio can be expressed as

(A12) $$E\left(\frac{P_{n}}{P_{w}}\right)\approx \frac{E\left(P_{n}\right)}{E\left(P_{w}\right)}-\frac{\mathrm{cov}\left(P_{n},P_{w}\right)}{E^{2}\left(P_{w}\right)}+\frac{E\left(P_{n}\right)\sigma^{2}\left(P_{w}\right)}{E^{3}\left(P_{w}\right)}.$$

Substituting Equations (A9)–(A11) into (A12), when the frequency error cannot be ignored, the expectation of the power ratio is approximated as

(A13) $$E\left(\frac{P_{n}}{P_{w}}\right)\approx \frac{M\left(c/n_{0}\tau {\mathrm{sinc}}^{2}\left(\pi f_{e}\tau \right)+1\right)}{M+c/n_{0}\tau {\mathrm{sinc}}^{2}\left(\pi f_{e}T_{coh}\right)}.$$
